# Curcumin and selenium synergistically mitigate oxidative stress in white-feathered broilers

**DOI:** 10.3389/fvets.2025.1600466

**Published:** 2025-06-02

**Authors:** Zixuan He, Zhaoyan Lin, Ye Yan, Jiao Wang, Shizhong Zhang, Bohan Zheng, Xiaohong Huang

**Affiliations:** ^1^College of Animal Science, Fujian Agriculture and Forestry University, Fuzhou, China; ^2^University Key Laboratory for Integrated Chinese Traditional and Western Veterinary Medicine and Animal Healthcare in Fujian Province/Fujian Key Laboratory of Traditional Chinese Veterinary Medicine and Animal Health, Fujian Agriculture and Forestry University, Fuzhou, China; ^3^Institute of Animal Husbandry and Veterinary Medicine, Fujian Academy of Agricultural Sciences, Fuzhou, China

**Keywords:** oxidative stress, curcumin, selenium, drug combination, IGF-1/PI3K/AKT/mTOR pathway

## Abstract

**Introduction:**

Oxidative stress is closely linked to various diseases in chickens, representing an urgent concern that needs to be addressed in the poultry industry. Curcumin (CUR) and selenium (Se) are both recognized for their great antioxidant effects, however, the combination use of them in broilers has not been reported. This study aims to demonstrate the synergistic antioxidant effects of CUR and Se *in vitro* and *in vivo,* and to explore the underlying antioxidant mechanism.

**Methods:**

The experiments were conducted on the DF-1 cell line and 400 healthy male White-feathered Broilers, day old, weighing 43.89 ± 0.70 g. Hydrogen peroxide (H₂O₂) and dexamethasone (Dex) were used to conduct oxidative stress model.

**Results:**

The results demonstrated that CUR and Se synergistically enhanced the antioxidant capacity of DF-1 cells, with a combination index (CI) less than 1; next, CUR and Se increased the total antioxidant capacity and superoxide dismutase (SOD) activity, decreased lactate dehydrogenase (LDH) and malondialdehyde (MDA) levels in broiler liver and heart tissues, alleviated Dex-induced liver and heart injury and liver cell apoptosis in broilers; moreover, the protein expression of IGF-1, PI3K-p110β, phosphorylated AKT and phosphorylated mTOR in liver and heart tissues were increased after the combination treatment.

**Discussion:**

In conclusion, CUR and Se alleviate oxidative stress in White-feathered Broilers synergistically, and the synergistic antioxidant effects are related to IGF-1/PI3K/AKT/mTOR pathway.

## Introduction

Oxidative stress is a status of imbalance between oxidation and reduction, which is closely associated with a variety of diseases in broilers (1–4). Oxidative stress induces metabolite accumulation in poultry, this condition further leads to higher mortality rates, reduced egg production, diminished meat and egg quality, and compromised overall performance (5–8). There are several factors that can induce oxidative stress, such as abnormal temperature (9, 10), high density (11), long-distance transport (12), etc. In modern feeding management, different strategies have been used to mitigate the effects of oxidative stress in poultry, such as genetic selection for better breeding (13), enhancing management strategies to mitigate stress within the production environment (14), antioxidant supplementation (15) and nutritional interventions (16) to improve poultry resistance.

The combined use of drugs for the prevention and treatment of diseases has emerged as a new trend (17). The advantages of drug combination include: synergistic effects of drugs leading to enhanced therapeutic outcomes; a reduction in the dosage of individual drugs, thereby mitigating drug side effects; and minimizing or slowing down the development of drug resistance, among others (18).

Curcumin (CUR) is a phenolic compound extracted from *Curcuma longa L*, the multiple medical value of CUR has been explored for over 30 years (19). Studies have proved that CUR can enhance the immunity and antioxidant capacity of poultry, improve lipid metabolism and intestinal health (20–23), simultaneously, CUR can ameliorate ochratoxin A induced oxidative stress through kelchlike ECH-Associating protein 1 (Keap1)-nuclear factor erythroid-2 related factor 2 (Nrf2)-antioxidant response element (ARE) and aryl hydrocarbon receptor (AHR) pathways (24). Other studies have shown that CUR can activate the mitogen-activated protein kinase (MAPK)-Nrf2 signaling pathway of chicken embryonic fibroblast cells to relieve heat-induced oxidative stress (25). Despite its exceptional antioxidant capabilities, the clinical application of CUR is limited by its low solubility and bioavailability (26). Consequently, numerous innovative strategies have been proposed to ameliorate these limitations, such as the use of biocarriers (27), the construction of structural analogs (28), and combination therapies (29), etc.

Selenium (Se) is one of the essential trace elements, and is crucial for maintaining fundamental life processes. Studies have demonstrated that Se possesses antioxidant properties and serves as the active site in antioxidant enzymes, including glutathione peroxidase (GPX) (30–33). Researchers have elucidated that Se can mitigate the spleen toxicity induced by aflatoxin B1 in broilers through the inhibition of oxidative stress and the attenuation of excessive apoptosis (34). In addition to the application alone, Se can also be used in combination with other agents to produce synergistic effects. For example, dietary supplementation of sodium selenite and vitamin E (VE) in broilers under summer heat stress can significantly increase the activities of catalase (CAT), superoxide dismutase (SOD), and GPX compared with Se or VE alone (35).

The Insulin-like growth factor 1 (IGF-1)/Phosphatidylinositol-3-kinase (PI3K)/Protein kinase B (AKT)/Mammalian target of rapamycin (mTOR) signaling pathway is critical in various vital life functions such as antioxidation (36, 37), cell growth (38), proliferation (39), differentiation (40) and apoptosis (41, 42). Previous studies have shown that activation of IGF-1 can activate PI3K, AKT and mTOR, which then activate the antioxidant function of cells to resist oxidative stress (43–45).

In the present study, the DF-1 cell line and White-feathered Broilers were used to test the hypothesis that CUR and Se exert synergistic anti-oxidative effects in White-feathered Broilers *in vitro* and *in vivo*, and the underlying mechanism is related to the IGF-1/PI3K/AKT/mTOR pathway. The findings support the use of CUR and Se as a potential antioxidant therapy in poultry industry.

## Materials and methods

### Cell maintenance and drug treatments

The chicken embryo fibroblast cell line DF-1 (Cell bank of the Chinese Academy of Science, Beijing, China) was cultured in Dulbecco’s Modified Eagle Medium (DMEM, Life Technologies Inc., Carlsbad, CA, United States) supplemented with 10% fetal bovine serum (FBS, Life Technologies Inc., CA, United States), penicillin (100 units/mL) and streptomycin (0.1 mg/mL; Life Technologies Inc., CA, United States) at 37°C in an atmosphere containing 5% CO_2_.

CUR was purchased from MedChemExpress Inc. (HY-N0005, Monmouth Junction, NJ, United States), Sodium Selenite was purchased from Sigma-Aldrich, Inc. (214,485, ST Louis, Missouri, United States). DF-1 cells were treated with 1–35 μM CUR, 0.1–3.5 μM Se or the combination of CUR and Se for 24 h, then the drugs were removed, 100 μM H₂O₂ was added to the fresh culture medium and cells were treated for 2 h before conducting subsequent experiments.

### Animals and treatments

The animal experiment was reviewed and approved by the Animal Ethics Committee of Fujian Agriculture and Forestry University (approval code: PZCASFAFU24029), according to the guidelines for Laboratory Animal Use and Care from the Chinese Center for Disease Control and Prevention and the Rules for Medical Laboratory Animals (1998) from the Chinese Ministry of Health.

A total of 400 healthy male White-feathered Broilers, which were day old and had similar body weight (43.89 ± 0.70 g) were acquired from Fujian Shengnong Inc. (Fujian, China) and randomly divided into 5 groups with 10 replicates per group and 8 broilers per replicate. Broilers were, respectively, fed diets supplemented with 100 mg/kg CUR, 0.45 mg/kg Se, or the combination of 100 mg/kg CUR and 0.45 mg/kg Se from day old to day 42. Broilers that needed to be sacrificed were anesthetized and euthanized on day 42 using Thiopental Sodium injection (0.7 mL/kg). Grouping and treatment protocols were presented in Table 1. Broilers were given free access to water and feed. The basal diet was formulated according to the National standard of the People’s Republic of China “Compound Feed for Laying Hens and Broiler” (GB/T 5916–2020). The composition and nutritional level of the basal diet were shown in Supplementary Table S1.

**Table 1:** Grouping and feeding protocols for broilers.

Group^1^	Feed
Con	Basal diet
Dex	Basal diet
Dex + CUR	Basal diet supplemented with 100 mg/kg CUR
Dex + Se	Basal diet supplemented with 0.45 mg/kg Se
Dex + CUR + Se	Basal diet supplemented with 100 mg/kg CUR and 0.45 mg/kg Se

1 Con, control; Dex, dexamethasone; CUR, curcumin; Se, selenium.

On the 35 d, 37 d and 39 d of the experiment, 3 mg/kg Dexamethasone (Dex) was injected intramuscularly into chickens in all groups except the control group, and the chickens in the control group was injected with the same amount of normal saline (46–50). The average daily feed intake (ADFI) and average daily weight gain (ADG) of broilers were recorded, with the feed conversion ratio (FCR) calculated using the formula: FCR = ADFI/ADG.

Serum samples were collected at 35 d to detect serum liver and kidney biochemical indices, while liver and heart tissues were collected at 42 d to perform pathological section with hematoxylin and eosin (H&E) staining and subsequent experiments.

### Antioxidant capability assays

Ferric Ion Reducing Antioxidant Power (FRAP) Assay Kit, Total Antioxidant Capacity Assay Kit with ABTS method (ABTS), SOD Activity Assay Kit, Malondialdehyde (MDA) Assay Kit (Beyotime Biotechnology Inc., Shanghai, China) and Lactate Dehydrogenase (LDH) Assay Kit (Nanjing jiancheng bioengineering Inc., Jiangsu, China) were used for detecting antioxidant capability of cells or tissues following the manufacturer’s instructions. Briefly, cells or tissues were homogenized using an ultrasonic grinder, after which the supernatant was collected into a 96-well plate and the appropriate reagents were added. The results were read using a microplate reader (Molecular Devices, 22,202-SANGMSMA1, Shanghai, China). The experiments were carried out in triplicate.

### Reactive oxygen species assay

Intracellular ROS levels were detected by DCFH-DA probe (Beyotime Biotechnology Inc., Shanghai, China) following the manufacturer’s instructions. Briefly, 1 × 10^6^ cells were plated in 6-well plates, the probe was diluted with a serum-free medium (1: 1,000), then cells were mixed with the probe and incubated at 37°C for 25 min in dark. The experiments were carried out in triplicate and the images were captured with a fluorescence microscope (IRX50, Sunny Optical Technology Inc., Jiangsu, China). Fluorescence intensity was measured using the Image J (Version 1.8.0, National Institutes of Health, United States) software.

### Serum liver and kidney biochemical indices assay

Serum liver and kidney biochemical indexes were determined by Shanghai Zhucai Biological Inc. (Shanghai, China). The levels of alanine aminotransferase (ALT), aspartate aminotransferase (AST), serum creatinine (CREA) and blood urea nitrogen (BUN) in serum were detected by automatic biochemical analyzer (Chemray240, Shenzhen Leidu Life Technology Inc., Shenzhen, China). The experiments were carried out in triplicate.

### Deoxynucleotidyl transferase-mediated dUTP nick end labeling assay

The TUNEL assay was performed to detect cell apoptosis. Briefly, liver samples were fixated in 4% paraformaldehyde for 1 h, and then were stained with TUNEL Apoptosis Assay Kit (abs50047, Absin, Shanghai, China) following the manufacturer’s instructions. Then the slides were mounted using a DAPI-containing medium (P0131, Beyotime Biotechnology Inc., Shanghai, China). Images were captured with a confocal laser scanning microscope (STELLARIS 5, Leica Inc., Wetzlar, Germany), six photographs were taken for each group. The number of apoptotic cells and the total number of cells in the visual field were counted by the ImageJ (Version 1.8.0, National Institutes of Health, United States) software, respectively, and then the apoptosis ratio was calculated using the equation: number of apoptotic cells/numbers of total cells × 100%.

### Transcriptome sequencing assay

The transcriptome sequencing assay was done by Majorbio Inc. (Shanghai, China). Total RNA was extracted from the tissue using TRIzol® reagent according the manufacturer’s instructions. RNA purification, reverse transcription, library construction and sequencing were performed at Shanghai Majorbio Bio-pharm Biotechnology Inc. (Shanghai, China) according to the manufacturer’s instructions (Illumina, San Diego, CA). To identify differential expression genes (DEGs) between two different samples, the expression level of each transcript was calculated according to the transcripts per million reads (TPM) method. Sequencing was performed on NovaSeqXPlus platform. The experiments were carried out in triplicate. The transcriptome sequencing data reported in this paper have been submitted to sequence read archive (SRA) database of NCBI and have been assigned the accession number PRJNA1192045.

### Immunohistochemistry assay

The tissue samples were fixed in 4% formaldehyde at pH 7.4 and 4°C for 48 h. After dehydration, paraffin wax was embedded and the tissues were sliced at a thickness of 4 μm. After deparaffination and antigen retrieval with EDTA antigen repair solution (MVS-0099, Maixin Biotechnology Inc., Fuzhou, China), sections were incubated with primary antibodies: IGF-1 (28530-1-AP, Proteintech Inc., Wuhan, China, 1: 200), PI3K-p110β (67121-1-Ig, Proteintech Group Inc., Wuhan, China, 1: 300 for liver, 1: 800 for heart), Phospho-AKT (66444-1-Ig, Proteintech Inc., Wuhan, China, 1: 200 for liver, 1: 800 for heart), and Phospho-mTOR (67778-1-Ig, Proteintech Inc., Wuhan, China, 1: 50) at 4°C overnight, followed by incubation of biotinylated secondary antibodies at 37°C for 1 h. Sections were stained with diaminobenzidine (DAB-1031, Maixin Biotechnology Inc., Fuzhou, China) and counterstained with hematoxylin (G1004, Servicebio Inc., Wuhan, China). Images were captured with a bright field digital microscope. The experiments were carried out in triplicate.

### Statistical analysis

The combination index (CI) was calculated to examine the interaction between CUR and Se by Compusyn software (version 1.0, Inc., Paramus, NJ, United States). CI values 1, <1, and >1 indicated an additive effect, synergism, or antagonism, respectively.

Statistical analysis was performed using GraphPad Prism10 software (version 10, GraphPad Software Inc., San Diego, CA, United States) and SPSS 26.0 software (version 26.0, IBM Inc., NY, United States). A two-tailed unpaired t-test with Welch’s correction was applied when the variances of two groups were proven equal by the F test. A *p* value of 0.05 or less indicated statistical significance.

## Results

### CUR and Se emerge synergistical antioxidant effect on DF-1 cells

In order to assess the combined effect of CUR and Se, different doses and ratio of CUR and Se were applied on DF-1 cells, and FRAP assays were performed, then the CI value was calculated based on the antioxidant results (Table 2). As the results shown, CUR and Se achieved synergistic effects at specific concentrations and varying ratios, the CI value reached its minimum at CUR 2.5 μM and Se 0.25 μM, indicating a better ratio of CUR to Se is 10:1.

**Table 2:** CI values of Cur and Se at different ratios and concentrations.

The proportion of combined drugs (CUR: Se)	CUR supplemental amount, μM	Se supplemental amount, μM	CI Value	The effect of combined drugs
5:1	1	0.2	1.99320	Antagonism
	2	0.4	2.44231	Antagonism
	2.5	0.5	0.57675	Synergy
	5	1	0.55580	Synergy
	10	2	1.13825	Antagonism
10:1	1	0.1	11496.3	Antagonism
	1.5	0.15	102.828	Antagonism
	2	0.2	538	Antagonism
	2.5	0.25	0.16236	Synergy
	5	0.5	0.61826	Synergy
	10	1	0.51245	Synergy
	15	1.5	2.12352	Antagonism
	20	2	3.02882	Antagonism
	25	2.5	11.3816	Antagonism
	30	3	745.668	Antagonism
	35	3.5	2152.38	Antagonism
20:1	2	0.1	0.20100	Synergy
	4	0.2	0.82056	Synergy
	5	0.25	0.45824	Synergy
	10	0.5	62.8905	Antagonism
	20	1	15.9132	Antagonism

1 The total antioxidant capacity of CUR and Se at different proportions and concentrations was determined by FRAP method, and the CI values were calculated by Compusyn software.

2 CUR, curcumin; Se, selenium.

**Figure 1:**
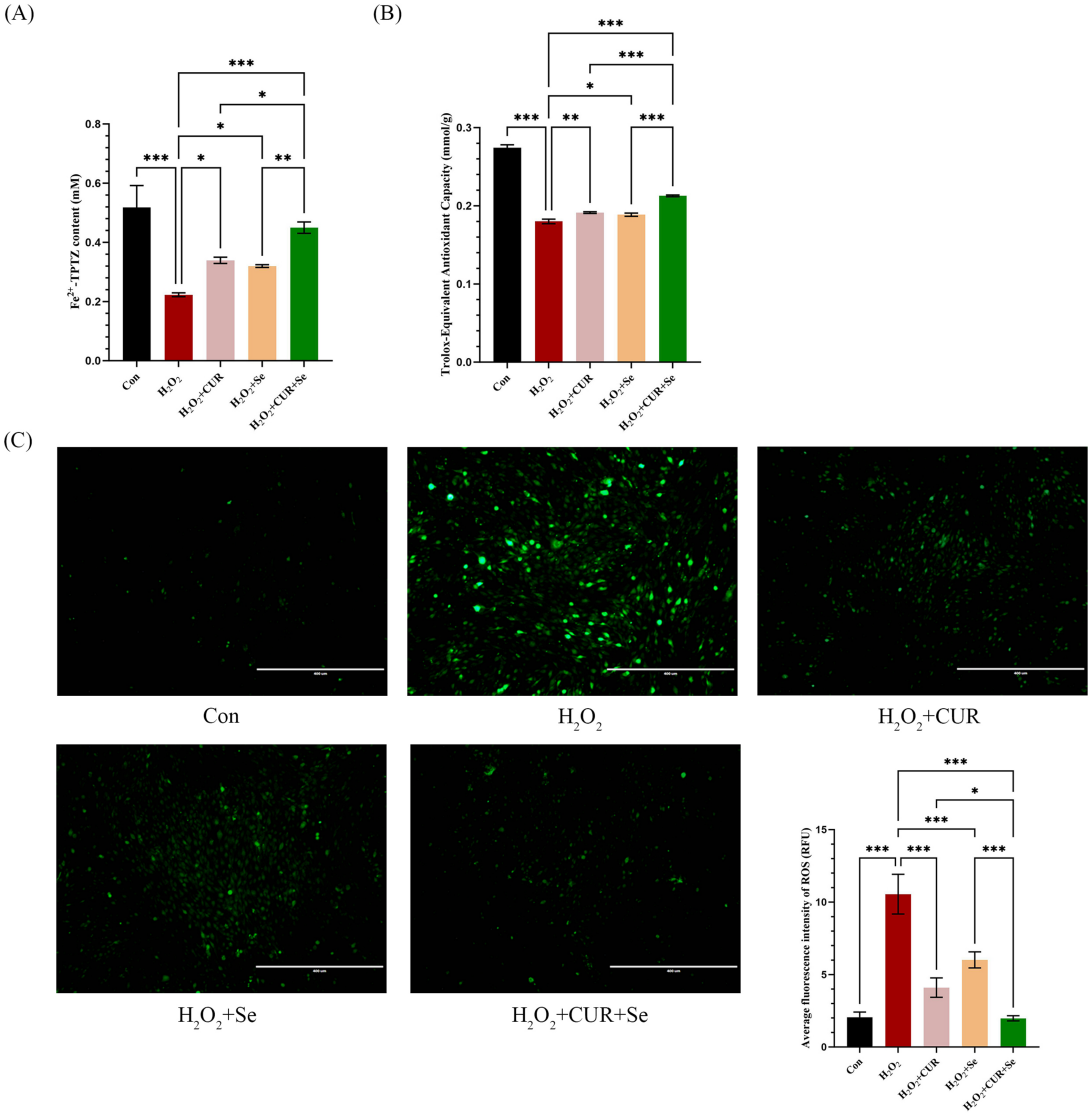
Cur and Se emerge antioxidant effect on DF-1 cells synergistically. DF-1 cells were plated in six-well plates and treated with 2.5 μM CUR and 0.25 μM Se in the medium for 24 h, then drugs were removed, 100 μM H₂O₂ was added to the culture medium for 2 h, after that, the FRAP **(A)**, ABTS **(B)** and ROS **(C)** assays were conducted following the manufacturer’s instructions. Data are representative of three independent experiments. Bar: 400 μm. *p < 0.05; ***p < 0.001. Con, control; Dex, dexamethasone; CUR, curcumin; Se, selenium.

Then, the FRAP, ABTS and ROS assays of DF-1 cells under these doses of CUR and Se were conducted to confirm the synergistic antioxidant effects (Figure 1). As the results shown, compared to the control group, H_2_O_2_ significantly decreased the cell total antioxidant ability, and increased the cellular ROS level (*p* < 0.001); CUR or Se alone treatments rescued the cells from the oxidative stress to a certain extent (*p* < 0.05), nevertheless, the combination treatment of CUR and Se significantly improved the antioxidant capability of DF-1 cells and reduced the production of ROS, compared with H_2_O_2_, CUR or Se groups (*p* < 0.05). These results demonstrated that CUR and Se emerged synergistic antioxidant effects *in vitro*.

### CUR and Se alleviate Dex induced hepatic and cardiac damage in broilers

To investigate the combined antioxidant effects of CUR and Se *in vivo*, a feeding trial with broilers was conducted, and an *in vivo* model of oxidative stress was established. There were no significant differences in FCR, ADG, ADFI or serum biochemical indices among all the groups (Supplementary Table S2; Supplementary Figure S1), indicating that CUR and Se did not have a notable impact on the growth performance or biochemical indices of broilers.

**Figure 2:**
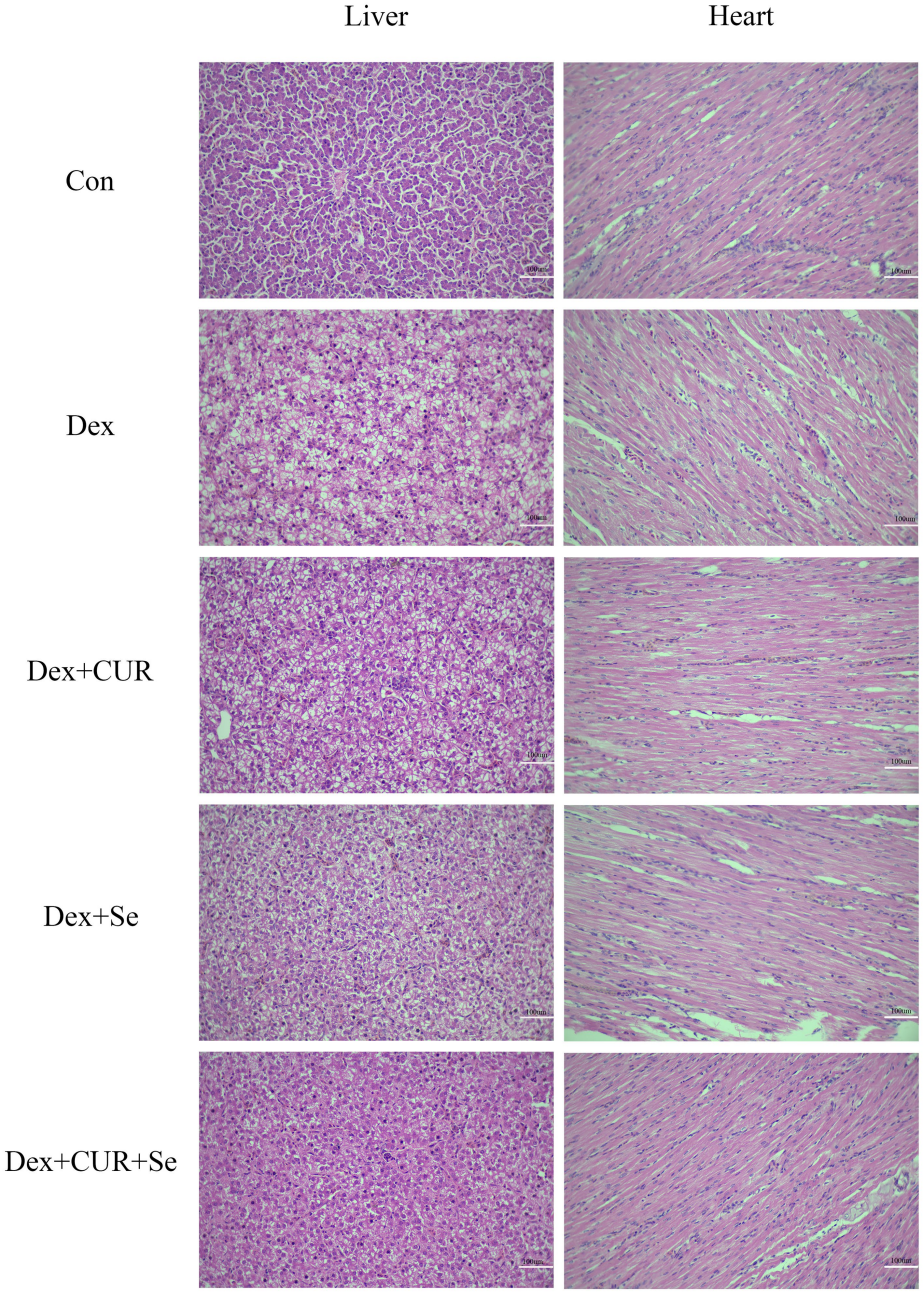
CUR and Se alleviate Dex induced hepatic and cardiac damage in broilers. Sections of liver and heart tissues from 42-day-old broilers, embedded in paraffin, were stained with hematoxylin and eosin (H&E) and examined under an optical microscope. Bar: 100 μm. Con, control; Dex, dexamethasone; CUR, curcumin; Se, selenium.

The liver and heart tissue sections were subjected to H&E staining (Figure 2). Compared with the control group, the liver of the Dex-treated group exhibited a substantial increase in the presence of vacuolar degeneration cells. This was predominantly characterized by the formation of transparent vacuoles within the cellular matrix, resulting in the displacement of cytoplasm to the periphery of the affected cells. Simultaneously, the number of vacuoles decreased in the drug-treated groups, especially in the combination treatment group. In addition, compared with the control group, the Dex-treated group also showed obvious hepatic cord disorder, resulting in no clear hepatic cord route, but the combination of drugs ameliorated this phenomenon. The results indicated that CUR and Se alleviated oxidative stress-induced liver damage.

A similar phenomenon was also observed in the heart tissues. Compared to the control group, the myocardial fibers in the Dex-treated group exhibited disarray and fragmentation, with an increase in intercellular spaces. Treatment with CUR or Se alone ameliorated this damage, but the combination of CUR and Se demonstrated a more pronounced beneficial effect.

### CUR and Se significantly ameliorate Dex-induced apoptosis in broilers’ liver tissues

**Figure 3:**
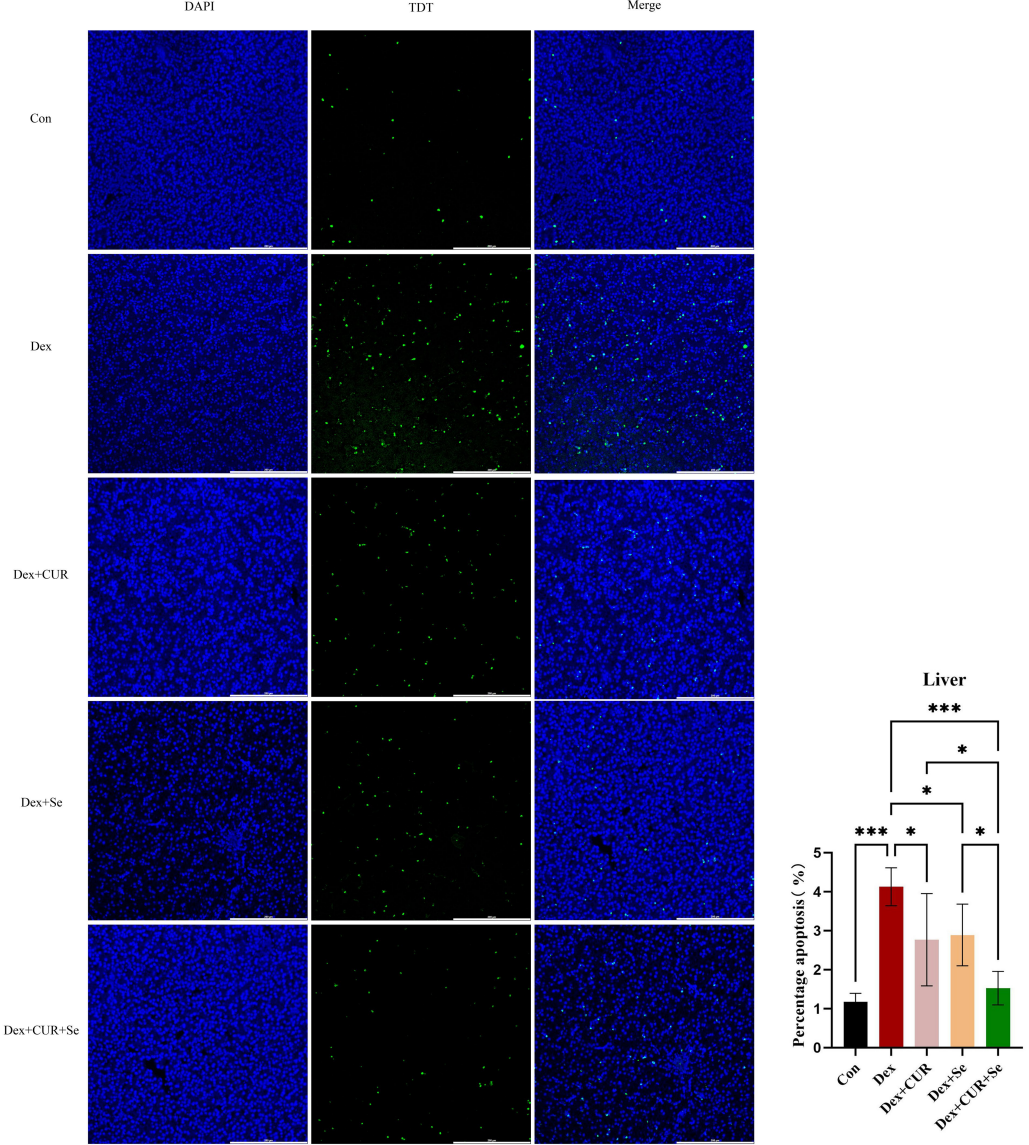
CUR and Se significantly ameliorate Dex-induced apoptosis in broilers’ liver tissues. Liver tissue sections were stained using TUNEL kits, the number of apoptotic cells and the total number of cells in the visual field were counted, respectively, and then the apoptosis ratio was calculated using the equation: number of apoptotic cells/numbers of total cells × 100%. Bar: 200 μm. Data are representative of six independent experiments. *p < 0.05; ***p < 0.001. Con, control; Dex, dexamethasone; CUR, curcumin; Se, selenium.

A TUNEL assay was performed to evaluate apoptosis level of the liver tissues. As shown in Figure 3, compared with the control group, the ratio of apoptotic cells in the Dex group increased significantly (*p* < 0.001), and compared with the Dex group, the ratio of apoptotic cells in all treatment groups was significantly reduced (*p* < 0.05). The level of apoptosis in the combination group is even lower compared to the monotherapy group (*p* < 0.05). These results indicated that combination of CUR and Se alleviated liver apoptosis under oxidative stress, thereby protecting the liver of broilers.

### CUR and Se enhance the antioxidant capacity of broilers’ liver and heart tissues

**Figure 4:**
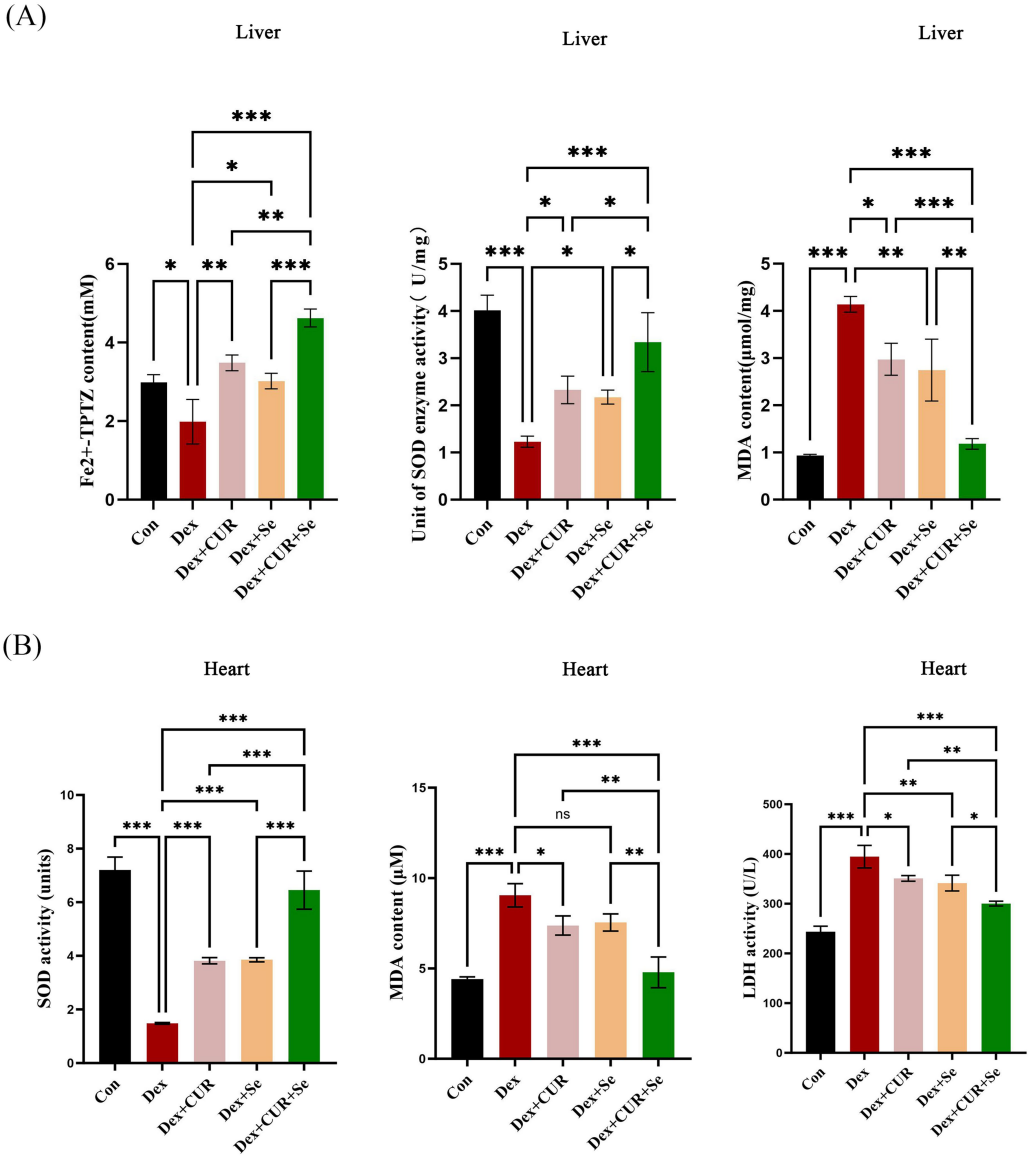
CUR and Se enhance the antioxidant capacity of broilers’ liver and heart tissues. The tissues were collected at 42 d, then FRAP, SOD and MDA assays were performed for the liver **(A)**, SOD, MDA and LDH assays were conducted for the heart. **(B)** Data are representative of three independent experiments. *p < 0.05; **p < 0.01; ***p < 0.001. Con, control; Dex, dexamethasone; CUR, curcumin; Se, selenium.

In order to investigate the synergistic antioxidant effect of CUR combined with Se *in vivo*, various assays were conducted in the liver and heart tissues. In the context of liver tissues (Figure 4A), when compared to the control group, the Dex group exhibited a significant reduction in total antioxidant capacity and SOD enzyme activity, along with a significant elevation in MDA content (*p* < 0.05); CUR and Se alone protected the liver form Dex to a certain extent (*p* < 0.05); while combined treatment emerged a better effect compared with that in the monotherapy groups (*p* < 0.05).

For the heart tissues (Figure 4B), compared with the control group, the activity of SOD in the Dex group was significantly decreased, while the MDA and LDH levels were significantly increased (*p* < 0.001). CUR and Se alone improved these indicators to a certain extent (*p* < 0.05), while combined treatment significantly relieved the damage caused by Dex compared with CUR or Se alone (*p* < 0.05). These results demonstrated that CUR and Se had synergistic antioxidant effects *in vivo.*

### The antioxidant mechanism of CUR and Se relates to IGF-1/PI3K/AKT/mTOR pathway

**Figure 5:**
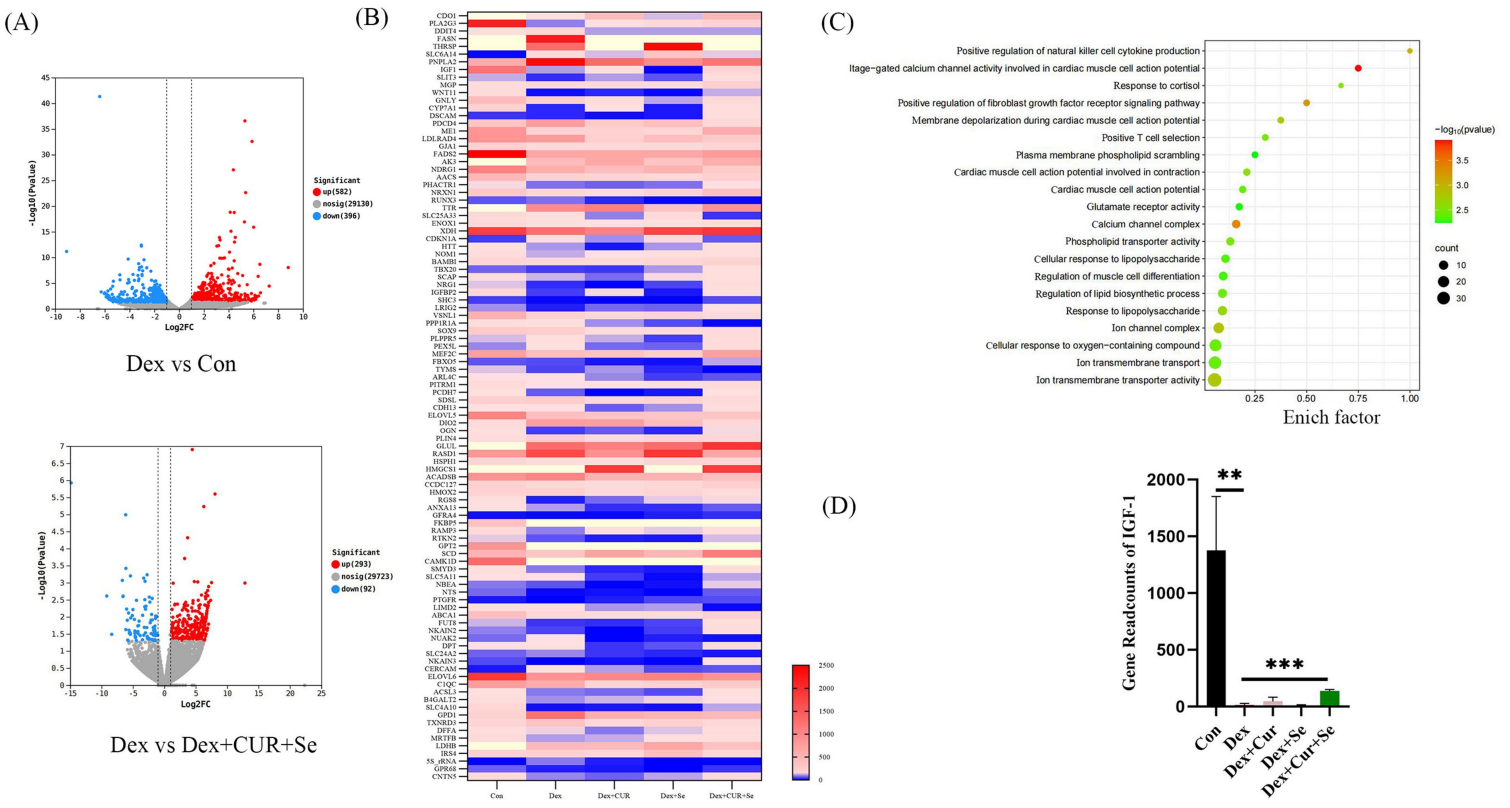
CUR and Se regulate signaling pathways associated with oxidative stress in broilers. **(A)** Gene expression differences among Control group, Dex group, Dex + CUR + Se group. **(B)** One hundred differentially expressed genes between the Dex group and the Dex + CUR + Se group. **(C)** The GO gene enrichment analysis results. **(D)** The gene readcounts of IGF-1 among groups. Data are representative of three independent experiments. **p < 0.01; ***p < 0.001. Con, control; Dex, dexamethasone; CUR, curcumin; Se, selenium.

To explore the underlying mechanism involved in the synergistical antioxidant effect of CUR and Se, a transcriptome sequencing assay of liver tissues were conducted (Figure 5). A total of 30,108 genes were detected, there were 582 genes up-regulated and 396 genes down-regulated in the Dex group compared with the control group, while when compared with the combination group, 293 genes were up-regulated and 92 genes were down-regulated in the Dex group (Figure 5A). Then, one hundred differentially expressed genes between the Dex group and the combination group were analyzed (Figure 5B). Meanwhile, the GO gene enrichment analysis revealed that the altered genes were enriched in multiple signaling pathways related to oxidative stress (Figure 5C). Among these differentially expressed genes, we noticed that IGF-1 was significantly down-regulated in the Dex group compared with that in the control group (*p* < 0.01), and up-regulated in the combination group compared with that in the Dex group (*p* < 0.001), as shown in Figure 5D.

**Figure 6:**
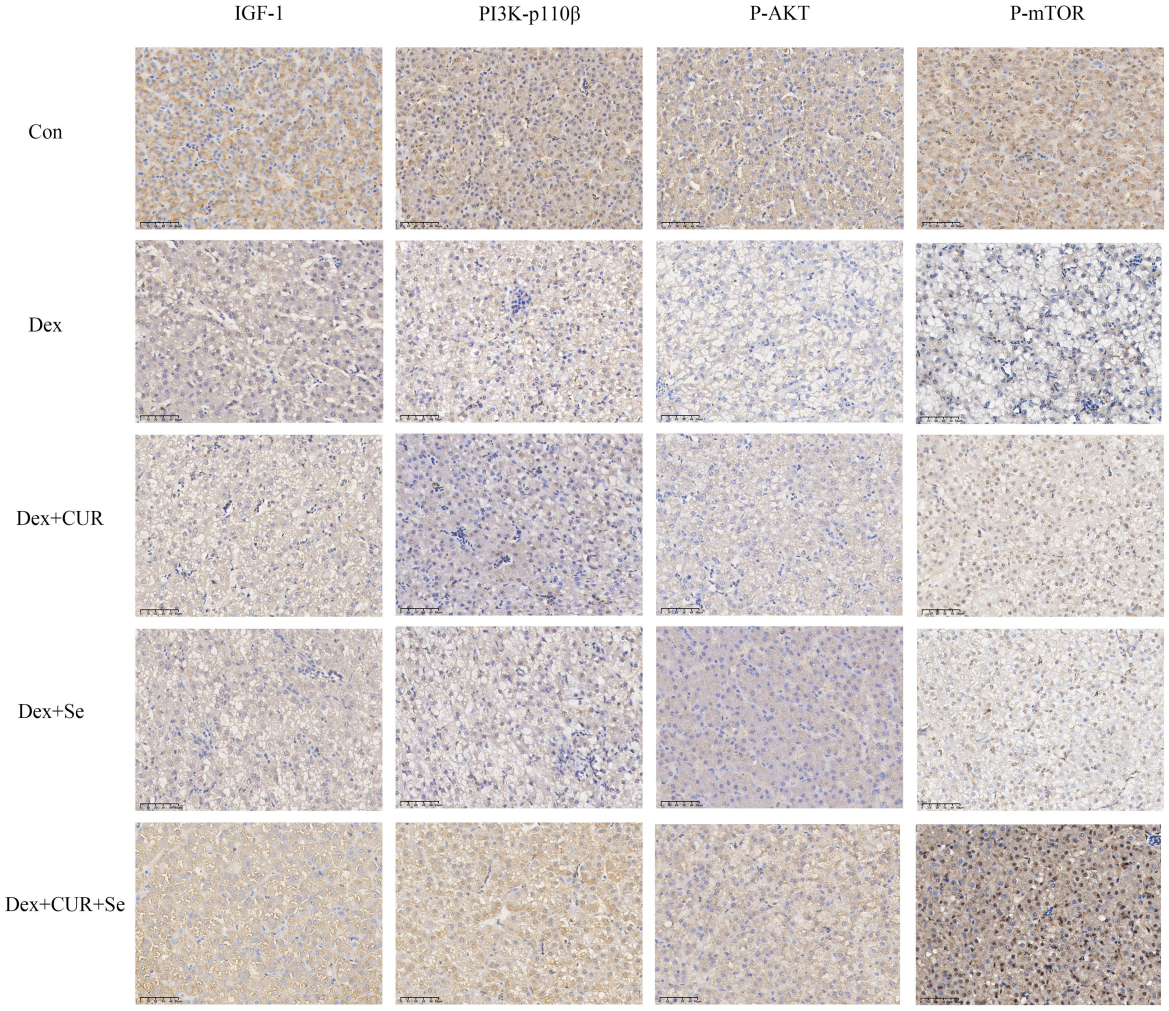
The IHC results indicated that IGF-1/PI3K/AKT/mTOR signaling pathway in broilers’ liver is activated by CUR and Se. Bar: 50 μm. Data are representative of six independent experiments. Con, control; Dex, dexamethasone; CUR, curcumin; Se, selenium.

**Figure 7:**
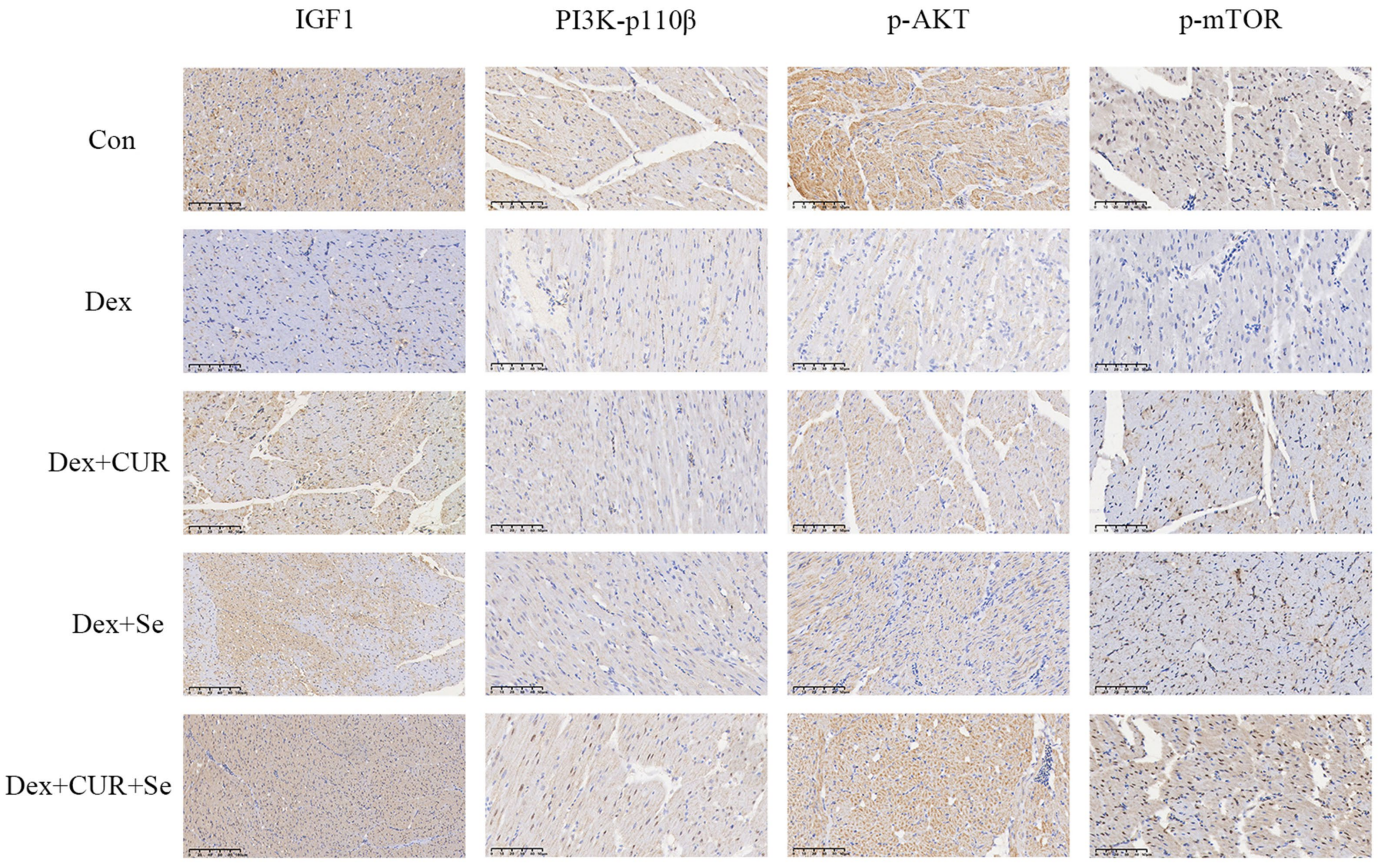
The IHC results indicated that IGF-1/PI3K/AKT/mTOR signaling pathway in broilers’ heart is activated by CUR and Se. Bar: 50 μm. Data are representative of six independent experiments. Con, control; Dex, dexamethasone; CUR, curcumin; Se, selenium.

Next, to further confirm the transcriptome sequencing results, the expression of IGF-1 related proteins in liver and heart tissues were detected by IHC. Consequently, the hepatic expression levels of IGF-1, PI3K-p110β, p-AKT, and p-mTOR were highest in the control group, followed by the combination group, with the Dex group exhibiting the lowest levels (Figure 6). In cardiac tissues, a comparable trend of alterations was observed (Figure 7).

## Discussion

Oxidative stress is closely associated with a variety of diseases in poultry, such as ascites syndrome in broilers (51), temperature stress (52), and egg-laying stress in laying hens (53), etc. The disease process is accompanied by oxidative stress, and the state of oxidative stress can further promote the progression of the disease (54). In the present study, H_2_O_2_ and Dex were selected as the inducer of oxidative stress, which is consistent with some previous studies (47, 48, 55). However, other studies have employed different types of agents to establish oxidative stress models in poultry, such as Diquat (56) or Decabromodiphenylether (57). These various inducers may simulate the oxidative stress status of chickens under real farming conditions in different aspects. Future studies could comprehensively evaluate the synergistic antioxidant effects of CUR and Se by employing different methods of inducing oxidative stress.

A previous study has showed that dietary supplementation of 50, 100 and 150 mg/kg curcumin can alleviate Diquat-induced oxidative stress, and the recommended dosage of curcumin in diets of broilers is in the range of 100 ~ 150 mg/kg (56). Another study applied a combination of vitamin E and organic selenium in the basal diet of broilers, the drugs alleviated the negative effects of heat stress by improving antioxidant status, regulating cytokine response, and altering the ileal flora and its function (58). In the present study, a therapeutic strategy combining CUR and Se was proposed to counteract oxidative stress in poultry, and the synergistic effects of these two reagents were discovered. Then, antioxidant assays have confirmed that the combination of CUR and Se exerts a stronger antioxidant effect compared to their individual use *in vitro* and *in vivo*.

One of the advantages of drug combination therapy is the potential to reduce the dosage of individual drugs, thereby decreasing the toxicity associated with their use. Numerous studies have demonstrated that the biological activity and toxic dose range of selenium are relatively narrow (59–61). Therefore, the antioxidant function of Se may be limited by its dosage application. In this study, a drug combination approach was employed to enhance the antioxidant properties of CUR or Se while avoiding potential toxicity. Additionally, some studies have indicated that the safety profile of organic Se or nano Se is superior to that of inorganic Se (62, 63). Hence, in future studies, the use of inorganic Se or nano Se in combination with CUR may lead to more favorable outcomes.

Oxidative stress is considered to be a key mediator leading to liver injury and progression of pathological liver disease (64). In the present study, the liver transcriptome sequencing results showed that the underlying antioxidant mechanism of CUR and Se was related to multiple antioxidant-associated pathways. The activation of IGF-1 can activate PI3K, AKT and mTOR, thereby activating the antioxidant function of cells to resist oxidative stress and reduce cell apoptosis (43–45). Studies have shown that IGF-1/PI3K/AKT/mTOR signaling pathway is the main pathway for ROS to mediate oxidative stress, regulating oxidative stress response in an active form (65, 66). The results of this study also confirmed that CUR and Se significantly enhanced the expression of IGF-1/PI3K/p-AKT/p-mTOR in liver and heart tissue. These results suggested that the underlying antioxidant mechanism of CUR and Se was related to IGF-1/PI3K/AKT/mTOR pathway. Nevertheless, the current study has only investigated the protein expression at the IHC level. Future research should extend to include Western blotting analysis and gene knockout models to provide a more comprehensive understanding of the results.

In conclusion, this study demonstrated that CUR and Se have a synergistic effect in combating oxidative stress in broilers, and their antioxidant mechanism is associated with the IGF-1/PI3K/AKT/mTOR signaling pathway. Further research in this area is warranted.

## Data Availability

The datasets presented in this study can be found in online repositories. The names of the repository/repositories and accession number(s) can be found at: https://www.ncbi.nlm.nih.gov/, PRJNA1192045.
