# Correction: Curcumin and selenium synergistically mitigate oxidative stress in white-feathered broilers

**DOI:** 10.3389/fvets.2025.1650011

**Published:** 2025-07-10

**Authors:** Zixuan He, Zhaoyan Lin, Ye Yan, Jiao Wang, Shizhong Zhang, Bohan Zheng, Xiaohong Huang

**Affiliations:** ^1^College of Animal Science, Fujian Agriculture and Forestry University, Fuzhou, China; ^2^University Key Laboratory for Integrated Chinese Traditional and Western Veterinary Medicine and Animal Healthcare in Fujian Province/Fujian Key Laboratory of Traditional Chinese Veterinary Medicine and Animal Health, Fujian Agriculture and Forestry University, Fuzhou, China; ^3^Institute of Animal Husbandry and Veterinary Medicine, Fujian Academy of Agricultural Sciences, Fuzhou, China

**Keywords:** oxidative stress, curcumin, selenium, drug combination, IGF-1/PI3K/AKT/mTOR pathway

In the published article there were terminology errors that required amendment.

The term “active-resistive exercises (ARE)” was intended to be “antioxidant response element (ARE)”, and “account health rating (AHR)” was intended to be “aryl hydrocarbon receptor (AHR)”.

A correction has been made to **Introduction**, paragraph 3. This sentence previously stated:

“CUR can ameliorate ochratoxin A induced oxidative stress through kelchlike ECH-Associating protein 1 (Keap1)-nuclear factor erythroid-2 related factor 2 (Nrf2)-active-resistive exercises (ARE) and account health rating (AHR) pathways (24).”

The corrected sentence appears below:

“CUR can ameliorate ochratoxin A induced oxidative stress through kelchlike ECH-Associating protein 1 (Keap1)-nuclear factor erythroid-2 related factor 2 (Nrf2)-antioxidant response element (ARE) and aryl hydrocarbon receptor (AHR) pathways (24).”

The original article has been updated.

