# Supplementary material for: Curcumin and selenium synergistically mitigate oxidative stress in white-feathered broilers
Source: Front Vet Sci. 2025 Jun 2;12:1600466. doi: 10.3389/fvets.2025.1600466 (PMC12171304; doi:10.3389/fvets.2025.1600466)
Supplement: Supplementary file 1 [file Data_Sheet_1.docx]

**Supplementary files**

Table S1. Composition and nutrient levels of basal diets (air-dry basis)

| Item | 1 to 21 days of ages | 22 to 42 days of ages |
| --- | --- | --- |
| Ingredients (%) |  |  |
| Corn | 51.75 | 53.26 |
| Soybean meal | 34.73 | 31.37 |
| Extruded soybean | 3.00 | 6.20 |
| Fish meal | 2.00 | 0.00 |
| Soybean oil | 4.21 | 5.00 |
| Limestone | 1.22 | 1.15 |
| CaHPO4 | 1.53 | 1.56 |
| DL-Methionine | 0.26 | 0.16 |
| NaCl | 0.30 | 0.30 |
| Premix^1)^ | 1.00 | 1.00 |
| Nutrient levels |  |  |
| Metabolic energy (MJ·kg^-1^) | 12.56 | 12.97 |
| Crude protein (%) | 21.5 | 20 |
| Ca (%) | 1 | 0.9 |
| Total P (%) | 0.45 | 0.4 |
| Lysine (%) | 1.19 | 1.08 |
| Methionine(%) | 0.59 | 0.45 |
| Methionine + Cysteine (%) | 0.91 | 0.76 |
| Threonine (%) | 0.82 | 0.76 |

^1^The premix provides the following per kg of diets:(1 to 21 days of ages) vitamin A 9800 IU,vitamin D3 2780 IU,vitamin E 19.6 mg,vitamin K3 2.24 mg,vitamin B1 1.4 mg,vitamin B2 7 mg,vitamin B3 33.6 mg,vitamin B5 9.8 mg,vitamin B12 0.0168 mg,pyridoxine 3.36 mg,biotin 0.056 mg,folic acid 1.12 mg,choline 1300 mg,Cu 8 mg,Fe 100 mg,Mn 120 mg,Zn 100 mg,Se 0.3 mg,I 0.7 mg;(22 to 42 days of ages) vitamin A 7000 IU,vitamin D3 2700 IU,vitamin E 14 mg,vitamin K3 1.6 mg,vitamin B1 1 mg,vitamin B2 5 mg,vitamin B3 24 mg,vitamin B5 7 mg,vitamin B12 0.0168 mg,pyridoxine 2.40 mg,biotin 0.04 mg,folic acid 0.8 mg,choline 1000 mg,Cu 8 mg,Fe 80 mg,Mn 100 mg,Zn 80 mg,Se 0.3 mg,I 0.7 mg.

^2^Metabolic energy is the calculated value, while the others are the measured values.

Table S2. Dex, Dex and CUR, Dex and Se and their synergistic effect on FCR, ADG and ADFI

| Age | Index | Con | Dex | Dex + CUR | Dex + Se | Dex + CUR + Se |
| --- | --- | --- | --- | --- | --- | --- |
| 1~21 | ADG, g/d | 550.19±35.8 | 555.52±44.12 | 539.73±26.55 | 552.79±30.71 | 541.15±24.31 |
|  | ADFI, g/d | 699.15±36.22 | 718.9±39.06 | 690.57±39.79 | 715.49±34.65 | 699.75±29.71 |
|  | FCR | 1.27±0.04 | 1.3±0.05 | 1.28±0.04 | 1.3±0.04 | 1.29±0.03 |
| 22~35 | ADG, g/d | 1168.2±83.42 | 1120.25±137.25 | 1197.5±82.63 | 1140.68±137.36 | 1178.56±73.03 |
|  | ADFI, g/d | 1653.03±76.52 | 1604.43±132.1 | 1644.72±110.99 | 1640.54±119.46 | 11620.55±99.07 |
|  | FCR | 1.42±0.08 | 1.44±0.13 | 1.38±0.07 | 1.45±0.16 | 1.38±0.06 |
| 36~42 | ADG, g/d | 542.71±75.89 | 499.19±96.64 | 529.97±115.36 | 487.68±150.61 | 496.73±154.87 |
|  | ADFI, g/d | 1079.03±65.13 | 1041.21±75.12 | 1072.62±69.53 | 1083.43±74.48 | 1055.97±65.08 |
|  | FCR | 2.02±0.29 | 2.14±0.35 | 2.1±0.4 | 2.46±0.94 | 2.31±0.74 |

^1^Absence of different letters on the shoulder of bar represent non-significant difference among lines.

^2^Con = control; Dex = dexamethasone; CUR = curcumin; Se = selenium; ADG = average daily gain; ADFI = average daily feed intake; FCR = feed conversion ratio.


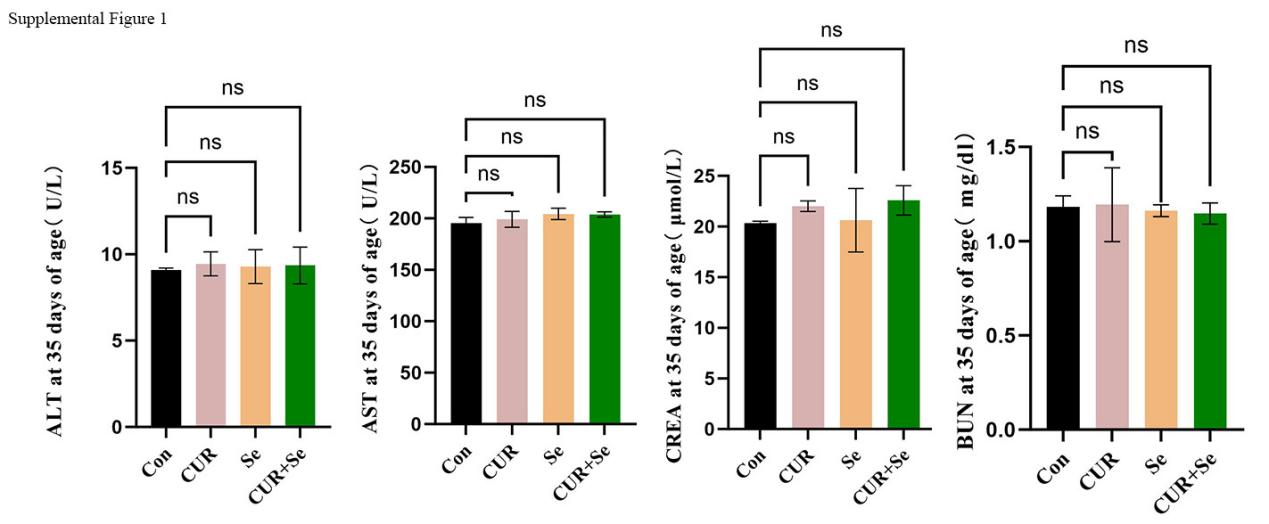


Fig. S1. CUR and Se Did Not Cause Toxicity to the Liver and Kidney of Broilers. Data are representative of three independent experiments. ns = non-significant; Con = control; Dex = dexamethasone; CUR = curcumin; Se = selenium; ALT = alanine aminotransferase; AST = aspartate aminotransferase; CREA = serum creatinine; BUN = blood urea nitrogen.
